# A simple and general strategy for generating frequency-anticorrelated photon pairs

**DOI:** 10.1038/srep24509

**Published:** 2016-04-18

**Authors:** Xin Zhang, Chang Xu, Zhongzhou Ren

**Affiliations:** 1 Department of Physics and Key Laboratory of Modern Acoustics, Nanjing University, Nanjing 210008, China; 2 Joint Center of Nuclear Science and Technology, Nanjing University, Nanjing 210093, China; 3 Center of Theoretical Nuclear Physics, National Laboratory of Heavy-Ion Accelerator, Lanzhou 730000, China

## Abstract

Currently, two-photon excitation microscopy is the method of choice for imaging living cells within thick specimen. A remaining problem for this technique is the damage caused by the high photon flux in the excitation region. To reduce the required flux, a promising solution is to use highly frequency-anticorrelated photon pairs, which are known to induce two-photon transitions much more efficiently. It is still an open question what the best scheme is for generating such photon pairs. Here we propose one simple general strategy for this task. As an example, we show explicitly that this general strategy can be realized faithfully within the widely applicable coherently pumped Jaynes-Cummings model. It is shown quantitatively that this strategy can generate highly frequency-anticorrelated photon pairs which can dramatically enhance two-photon excitation efficiency. We believe the proposed strategy can guide new designs for generating frequency-anticorrelated photon pairs.

We know from our childhood playing that when covering a lamp using our hand, red light comes out from the back. This is because inside tissues, red and infrared light can penetrate longer distances than other visible lights. In two-photon excitation microscopy^1^, red and infrared light is used to induce two-photon excitations of fluorescent compounds that absorb single photons in the visible range. Thanks to the much longer penetration depth of these photons, as well as several other unique features, two-photon excitation microscopy is right now the method of choice for *in vivo* imaging of living cells within thick specimen^2^,^3^. A remaining problem to be solved in two-photon excitation microscopy is the damage caused by the high photon flux in the excitation region, where high flux has to be used to compensate for the small cross section of two-photon excitation^4^,^5^. To reduce these potential damages to sensitive biological samples, a promising solution is to use highly frequency-anticorrelated photon pairs^6^. Such photon pairs have a sum-frequency (the total energy of the photon pair divided by Planck’s constant *h*) width which is much narrower than the frequency width of either photon. It is known that these photon pairs can induce two-photon excitations much more efficiently than classical uncorrelated light^6^,^7^,^8^,^9^,^10^,^11^,^12^,^13^,^14^. Due to the much higher efficiency, much less photon flux is needed and the damage to sensitive biological samples can be drastically reduced.

A vital ingredient in this solution is the availability of frequency-anticorrelated photon pairs. At present, the most used method to generate them is through parametric down conversion^6^,^7^,^8^,^15^,^16^,^17^, and preliminary experiments on two-photon excitation induced fluorescence have been performed using thus-generated photon pairs^6^. Also, it is proposed that uncorrelated photon pairs can be converted into frequency-anticorrelated photon pairs using some ingeniously designed schemes^18^,^19^. Nevertheless, it is still an open question what scheme is optimal for generating such photon pairs for use in microscopy applications. It would be desirable if there is a general strategy for generating frequency-anticorrelated photon pairs that does not assume much details of the physical realization. This would effectively offer many alternatives corresponding to different actual realizations, from which the optimal scheme can be picked. Such a strategy could also serve to inspire new schemes for generating frequency-anticorrelated photon pairs.

In this paper we propose one simple general such strategy. This strategy is to find or design the following generic scenario: an initial state A, which is otherwise stable, is coherently and weakly coupled to state B, which decays quickly through single photon-emission to state C. State C in turn decays quickly through single photon-emission to a final stable state D. More succinctly, this can be written as “
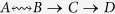
”, where “
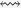
” denotes a weak coherent coupling, while “→” denotes fast irreversible single-photon emission (cf. Fig. 1). This strategy is robust in the sense that, guaranteed by generally valid physical principles such as energy conservation and the time-energy uncertainty relation, the photon pairs thus generated will be frequency-anticorrelated. As an explicit example, we show that this simple strategy can be realized faithfully in the widely applicable coherently pumped Jaynes-Cummings (JC) model^20^. Furthermore, the wave function of the outgoing photon pair is derived analytically and it shows quantitatively that the photon pair can possess pronounced frequency-anticorrelation. Finally, through a simple modeling of the processes of two-photon excitation induced fluorescence, we show that these frequency-anticorrelated photon pairs can give a large enhancement in two-photon excitation induced fluorescence compared to uncorrelated photon pairs. Our results thus show that, the proposed simple general strategy is capable of generating highly frequency-anticorrelated photon pairs that can dramatically enhance two-photon excitation efficiency, which could be crucial for low-flux two-photon microscopy applications.

## Results

### Intuitive justification of the proposed strategy

**Figure 1:**
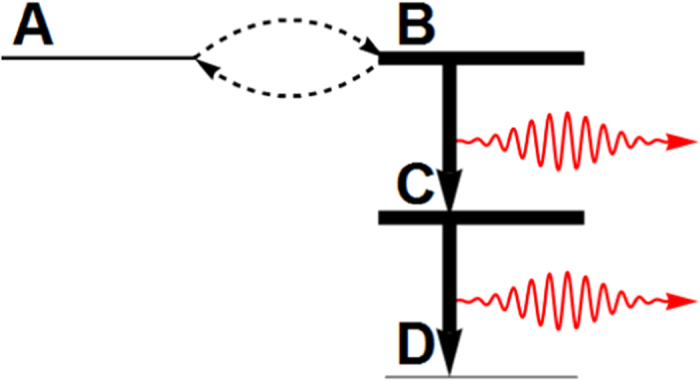
Proposed general strategy for generating frequency-anticorrelated photon pairs. This strategy is to find or design the depicted scenario: an initial state *A*, which is otherwise stable, is coupled coherently and weakly with state *B*, which decays quickly through single-photon emission to state *C*, which also decays quickly through single-photon emission to a final, stable state *D*. As explained in the text, guaranteed by generally valid physical principles, the photon pair thus generated will be frequency-anticorrelated.

Using the proposed strategy “
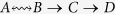
” the photon pair generated is frequency-anticorrelated. This can be understood intuitively as follows (cf. Fig. 1). On the one hand, the frequency width of either photon is basically determined by the frequency width of the states B and C. Since both of these states decay very fast, due to the time-energy uncertainty relation, their widths must be quite large, and consequently the emitted photons must have quite large frequency widths. On the other hand, we consider the sum frequency of the photon pair, which must be equal to the energy of the initial state A relative to the final state D (setting Planck’s constant *h* to unity) due to energy conservation. Since state D is stable and thus has zero width, the sum-frequency width is solely determined by the width of state A. But this width must be very narrow since state A only decays slowly via its weak coupling to state B and thus has a long lifetime. So to sum up, under the scenario described by the proposed strategy, the frequency width of either photon must be quite large while the sum-frequency width has to be narrow. Thus the photon pair is by definition frequency-anticorrelated. This is the main idea we wish to communicate through the present report.

### A faithful realization of the proposed strategy in the coherently pumped JC model

**Figure 2:**
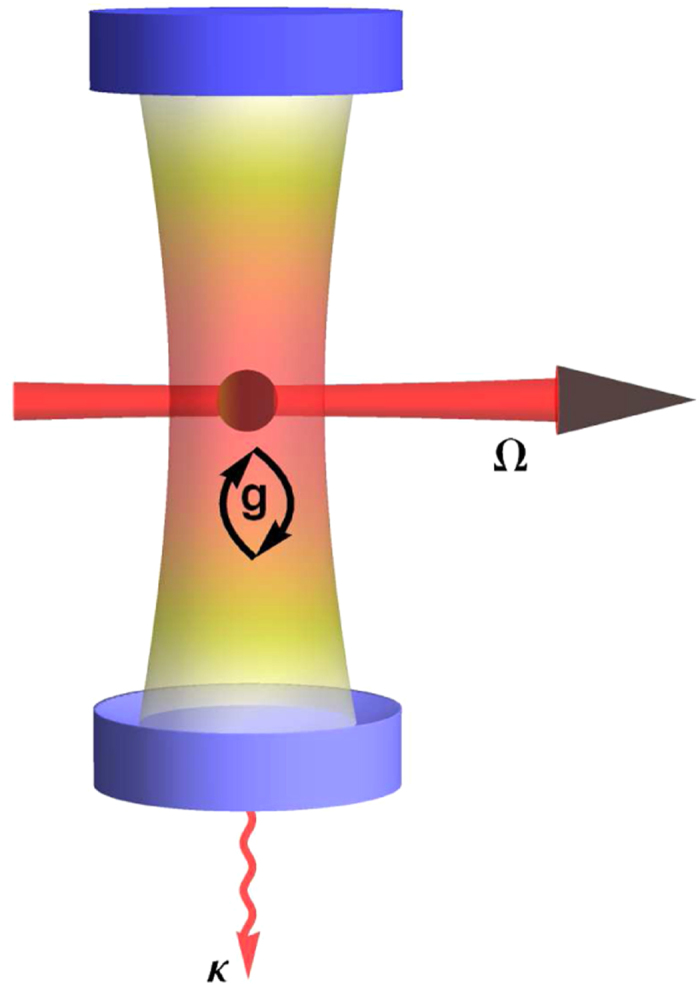
Schematic depiction of the coherently pumped JC model. A photon cavity is formed by two high-quality mirrors facing each other. The photon mode supported by the cavity interacts coherently with the two-level system (the sphere in the middle) with a strength characterized by *g*. The two-level system is coherently pumped by a laser beam with a pumping strength characterized by Ω. The upper mirror is a high reflector while the lower mirror is slightly transmitting, which gives rise to the finite cavity decay rate *κ*. Note that this figure is intended to be a schematic depiction for all equivalent systems describable by the coherently pumped JC model (equation (1)). This model can be used to realize faithfully the proposed general strategy.

The JC model^20^ is the most important, most widely applicable and possibly the simplest model in the area known as cavity quantum electrodynamics (cavity QED)^21^,^22^,^23^,^24^,^25^,^26^,^27^. The proposed simple general strategy “
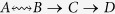
” can be realized faithfully in the widely applicable coherently pumped JC model. A schematic depiction of the basic scenario of this model is given in Fig. 2. It describes a single-mode photon field interacting with a two-level system (TLS), which is pumped coherently by a laser. Note that in Fig. 2 a finite cavity decay characterized by the decay rate *κ* is also included. This decay arises from the coupling of the intra-cavity field with the extra-cavity photon modes and shall be crucial in generating the outgoing photon pairs. This model is described by the following Hamiltonian (written in the rotating frame of the pumping laser):


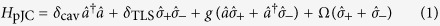


where the first term is the free cavity-mode Hamiltonian, 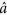 is the annihilation operator of the single-mode cavity field, 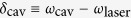 where *ω*_cav_ is the bare cavity resonance frequency and *ω*_laser_ is the laser frequency, the second term is the free TLS Hamiltonian, 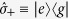, 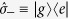 where |*e*〉 and |*g*〉 are the upper and lower states of the TLS, *δ*_TLS_ ≡ *ω*_TLS_ − *ω*_laser_ where *ω*_TLS_ is the bare frequency of the TLS, the third term is the coupling between the cavity field and the TLS where *g* is the coupling constant, the fourth term is the coherent pumping of the TLS by the laser^27^,^28^ where Ω characterizes the pumping strength.

In the strong coupling regime, i.e. *g* ≫ *κ*, by suitably choosing the system parameters, the above Hamiltonian can be equivalent to an effective Hamiltonian that, together with the cavity decay, matches precisely the proposed strategy. Consider the large detuning - strong pumping regime, i.e. |*δ*_cav_ − *δ*_TLS_| ≫ *g* and Ω ≫ *g*. In this regime the TLS and the cavity field is essentially decoupled from each other. Now the eigenstates and eigenenergies are determined to the zeroth-order by


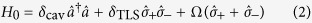


to be


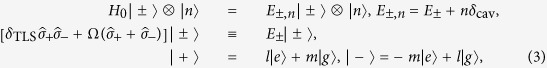


where *l* and *m* are real expansion coefficients, and |*n*〉 denotes an n-photon Fock state that satisfies 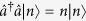. Hereafter we shall use the notation |±*n*〉 for 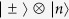. If the eigenstate |+2〉 is populated, due to cavity dissipation it will decay irreversibly through the route |+2〉 → |+1〉 → |+0〉, emitting one photon in each step. Since |+0〉 is stable, this fulfills the latter part of the strategy “*B* → *C* → *D*” (cf. Fig. 1).

The whole strategy would be fulfilled if |−0〉, which is otherwise stable as required in the proposed strategy, is coupled weakly and coherently to |+2〉. Luckily this can also be engineered. For parameter combinations under which |−0〉 and |+2〉 are near degenerate, a weak effective coupling between these two states is induced by the bare atom-cavity coupling. More quantitatively, when |−0〉 and |+2〉 is near degenerate an effective Hamiltonian for the system can be derived using the adiabatic elimination method^29^,^30^,^31^ to be:


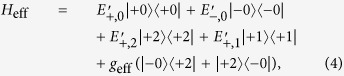


where 
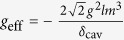
, while the primed energies 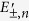 differ slightly from the corresponding unprimed ones (cf. equations (3) and (8)). As can be seen from the last term in *H*_eff_, |−0〉 and |+2〉 are coherently coupled to each other. For suitably chosen parameters, *g*_eff_ can be much smaller than the cavity decay rate *κ* (the quantitative requirement on how small *g*_eff_ must be shall become clear when we have the analytical wave function of the photon pair), thus fulfilling the remaining part (“
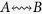
”) of the proposed strategy.

**Figure 3:**
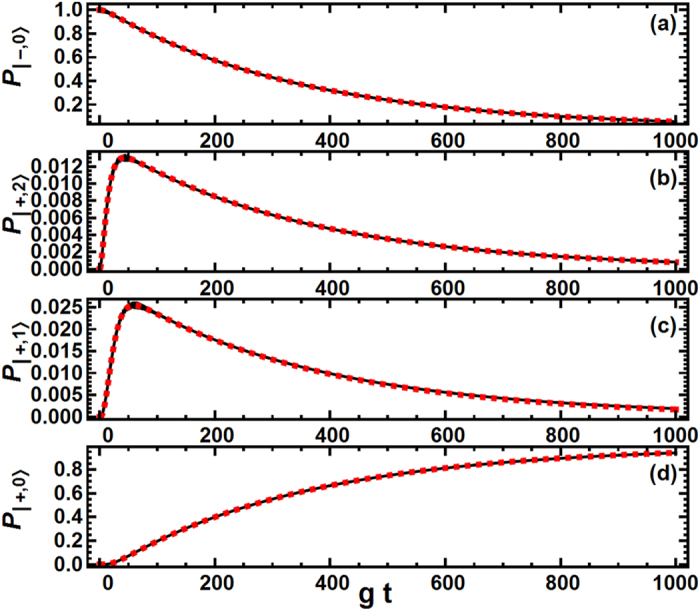
Justification of the effective Hamiltonian. Here we give a comparison of the population dynamics calculated according to respectively the original Hamiltonian *H*_pJC_ (equation (1)) and the effective Hamiltonian *H*_eff_ (equation (4)), in the presence of cavity decay. (**a**–**d**) Give the time-dependence of the occupations of the states |−0〉, |+2〉, |+1〉 and |+0〉, respectively. The original-Hamiltonian results are given by black solid lines, while the effective-Hamiltonian results are given by red dashed lines. As can be seen, *H*_pJC_ and *H*_eff_ give essentially indistinguishable results. The initial state is |−0〉. *g* = 1, *κ* = 0.1, Ω = 32, *δ*_cav_ = −34.46 and *δ*_TLS_ = 25.54 in equation (4). Calculational details are given in Methods. Note that it may seem very strange that the population would go spontaneously from the low energy state |−0〉 to the high energy state |+2〉. However, the extra energy is actually provided by the pumping laser. This process can be understood naturally using the dressed state picture. For an elucidating discussion on this point, we refer to ref. 37.

**Figure 4:**
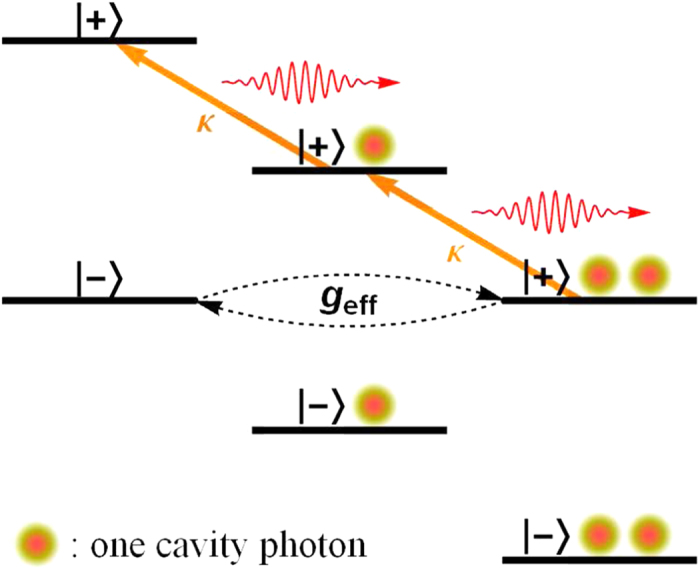
Realization of the proposed general strategy in the coherently pumped JC model. The proposed general strategy (cf. Fig. 1) can be realized faithfully in the coherently pumped JC model in the strong coupling - strong detuning - strong pumping regime. The levels drawn are the eigenstates |±*n*〉(*n* = 0, 1, 2) of the zeroth-order Hamiltonian (equation (2)). The initial state |−0〉 is coupled through the weak effective coupling to |+2〉, which decays quickly through single-photon emission to |+1〉, which decays quickly through single-photon emission to the stable state |+0〉. These match precisely with the proposed strategy. Note that this level scheme is drawn within the interaction picture of the laser (cf. equation (1)), and thus the energy scales are not in the optical domain but on the scale of (cf. equation (3)) the difference frequencies *δ*_TLS_, *δ*_cav_ and the Rabi frequency of the laser Ω. Within the large detuning - strong pumping regime as considered here, the two-photon resonance condition between |−0〉 and |+2〉 as shown can always be met by tuning *δ*_cav_ via changing the resonance frequency of the cavity (cf. equation (3)).

Now we only need to verify that this effective Hamiltonian indeed correctly describes the dynamics of the TLS-cavity system. We show in Fig. 3 for the typical parameter combination studied in this paper a comparison between the predictions given by the original Hamiltonian (equation (1)) and those given by this effective Hamiltonian (equation (4)) in the presence of cavity decay. In the figure we plot the evolutions of the occupation-probabilities for four states: |+0〉, |+1〉, |+2〉 and |−0〉. As can be seen, for all the four states the two curves to be compared are essentially indistinguishable. Thus the effective Hamiltonian describes the system dynamics correctly. To sum up, the proposed strategy “
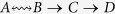
” can be realized faithfully using the coherently pumped JC model to be “
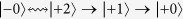
” by working in the strong coupling - large detuning - strong pumping regime and tuning the state |−0〉 and |+2〉 to be near degenerate. The realized scenario is depicted schematically in Fig. 4.

### Highly frequency-anticorrelated photon pairs

Now it is clear that the proposed general strategy can be realized faithfully in the coherently pumped JC model. Can the thus realized strategy indeed generate frequency-anticorrelated photon pairs? And if so, how pronounced is the frequency-anticorrelation?

To answer these questions, the wave function of the outgoing photons is needed. Physically, the generation of the outgoing photons results from the coupling of the cavity mode with the extra-cavity photon modes via the slightly transmitting mirror (cf. Fig. 2). The full Hamiltonian for the TLS-cavity system plus the extra-cavity photon modes is as follows^32^,^33^:


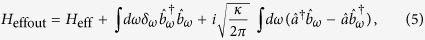


where the first term *H*_eff_ is the effective Hamiltonian of the TLS-cavity system given in equation (4), the second term is the free Hamiltonian of the extra-cavity photon modes, 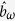 is the annihilation operator for the extra-cavity mode with frequency *ω* and obeys the standard commutation relation 
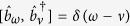
, 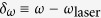, the third term is the coupling between the cavity mode and the extra-cavity photon modes where *κ* is the cavity decay rate. For simplicity and without loss of generality, we assume the system is initialized in the state |−0〉. The time-dependent Schrödinger equation can be solved analytically (see Methods) giving the following long-time asymptotic expression for the wave function (up to an irrelevant overall phase factor):


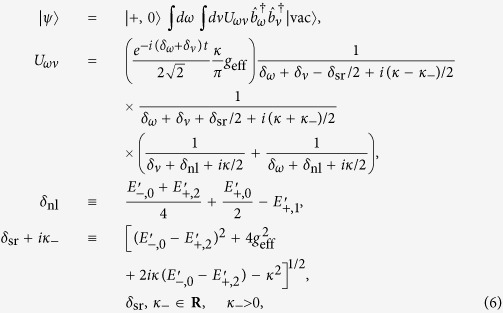


where |vac〉 denotes the vacuum of the extra-cavity photon modes, and here *δ*_*ω*_ means 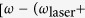

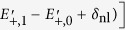
.

**Figure 5:**
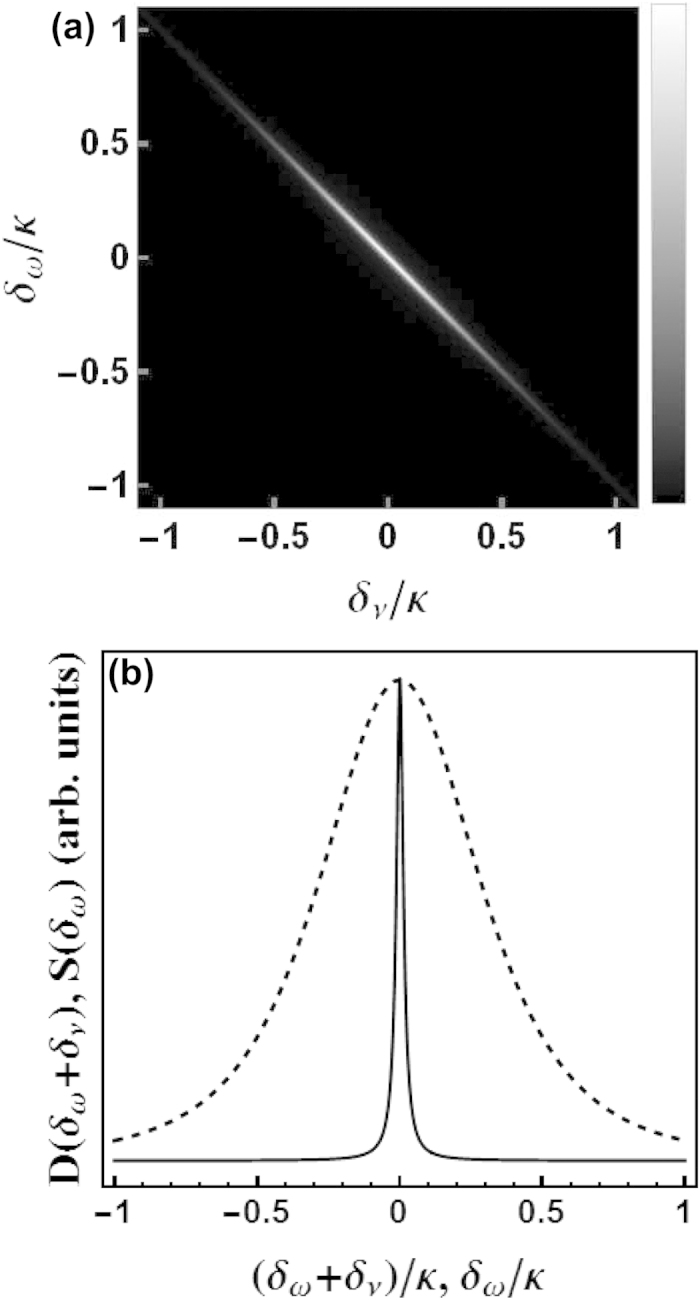
Pronounced frequency anticorrelation of the emitted photon pair. (**a**) The probability density (arbitrary units) for one photon to have frequency *δ*_*ω*_ and the other to have frequency *δ*_*v*_, for the photon pair generated using the coherently pumped JC model (equation (6)). Both axis are measured in units of the cavity decay rate *κ*. As can be seen, the probability distribution concentrates on a line described by *δ*_*ω*_ + *δ*_*v*_ = *constant*, which is just a defining feature for frequency anticorrelation. (**b**) Probability distribution (arbitrary units) of the sum frequency *D*(*δ*_*ω*_ + *δ*_*v*_) (solid) compared with that of the frequency of either photon regardless of the frequency of the other photon *S*(*δ*_*ω*_) (dashed), for the same photon pair as in (**a**). The heights of the two distributions are normalized to be the same at the center. The horizontal axis give the frequencies in units of the cavity decay rate *κ*. As can be seen, the sum-frequency distribution is much narrower than the frequency distribution of either photon. The photon pair is thus by definition highly frequency-anticorrelated. The parameters used are the same as in Fig. 3.

As can be seen from the above equation, the asymptotic wave function |*ψ*〉 contains only the component describing two photons occupying the extra-cavity modes. This means exactly one outgoing photon pair is generated. From the second term of the expression for *U*_*ωv*_, it can be seen that the photon pair has a sum-frequency width ~(*κ* − *κ*_−_). Since we work under the condition (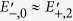), this width is essentially 
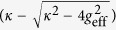
 (cf. equation (6)). Thus as long as 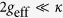, the sum-frequency width shall be much smaller than the width of either photon, *κ*, i.e., the photon pair shall be highly frequency-anticorrelated. A popular way to visualize frequency correlations is to plot the joint frequency distribution 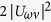, namely, the probability density for one photon to have frequency *δ*_*ω*_ and the other to have frequency *δ*_*v*_. This is shown in Fig. 5a for the typical parameter combination studied here. As can be seen, the probability distribution concentrates on a line described by *δ*_*ω*_ + *δ*_*v*_ = *constant*, which is just a definitional feature of frequency anticorrelation^17^. More directly, to confirm the existence of frequency-anticorrelation one can compare the sum-frequency distribution 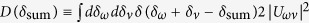 with the frequency distribution of either photon 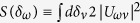. This is shown in Fig. 5b. As can be seen, the sum-frequency distribution is much narrower than the frequency distribution of either photon. The generated photon pair is thus indeed highly frequency-anticorrelated.

### Large enhancement in two-photon excitation induced fluorescence

Now it is clear that the general strategy as realized in the coherently pumped JC model is capable of generating highly frequency-anticorrelated photon pairs. Do these photon pairs indeed offer some enhancement in two-photon-excitation induced fluorescence compared with uncorrelated photons? And if so, how large can the enhancement be?

**Figure 6:**
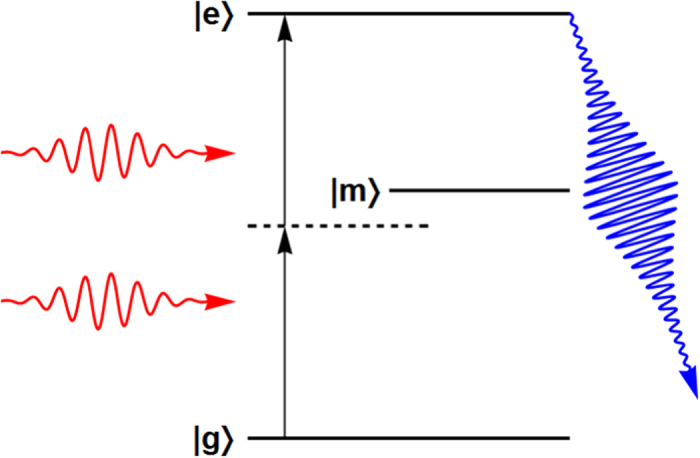
The three-level model used for estimating the amount of two-photon excitation induced fluorescence. The two incoming photons (represented by the red wiggly arrows) induce the two-photon transition from |*g*〉 to |*e*〉 via the far detuned intermediate state |*m*〉. The fluorescent photons (blue wiggly arrow) comes from the decay of the excited state |*e*〉. We use this simple model to compare the amount of two-photon excitation induced fluorescence due to the frequency-anticorrelated photon pair (equation (6)) and that due to an uncorrelated photon pair (equation (7)).

To answer these questions, we use a simple three-level model to simulate the processes of two-photon excitation induced fluorescence. The three-level model, as is commonly used in the analysis of two-photon excitation^34^,^35^, consists of a ground state |*g*〉, an intermediate state |*m*〉, and a final excited state |*e*〉 (cf. Fig. 6). The amount of fluorescence is given by the accumulative decay from the state |*e*〉 into modes other than the continuum modes carrying the incoming photon pairs. In the present work we restrict ourselves to a resonant two-photon excitation and a far detuned intermediate state. For the frequency-anticorrelated photon pair generated using the coherently pumped JC model, the initial condition is given by equation (6). For uncorrelated photon pairs, without loss of generality the initial condition can be written in the following product form^18^,^19^:


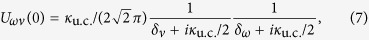


where 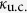 is the frequency width of either photon. Assuming the coupling between the incoming photon pair and the three-level system is weak enough, the back action on the wave function of the incoming photon pair can be ignored, and then the time-dependent Schrödinger equation can be solved analytically giving the amount of fluorescence. More detail is given in Methods.

**Figure 7:**
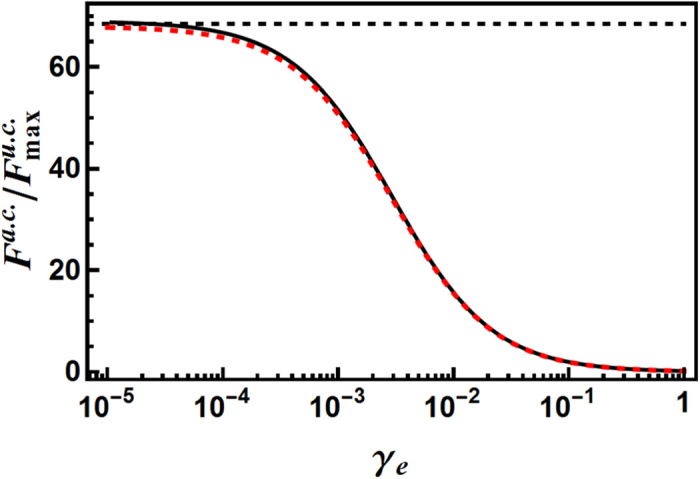
Comparison of two-photon excitation induced fluorescence. This figure gives the ratio of the amount of two-photon excitation induced fluorescence achieved by the frequency-anticorrelated photon pair (equation (6)) 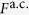 to the maximum achievable amount by the uncorrelated photon pair (equation (7)) 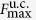, as a function of the decay constant *γ*_*e*_. *γ*_*e*_ characterizes the rate at which the excited state |*e*〉 (cf. Fig. 6) decays into the fluorescence photon modes (cf. Methods). Black dashed line: the estimated asymptotic enhancement factor 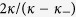. Red dashed line: the estimated enhancement factor as a function of *γ*_*e*_, 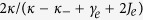. Black solid line: exact results (cf. Methods). As can be seen, when the spontaneous decay rate from the final state |*e*〉 is small, the frequency-anticorrelated photon pair can give a near seventy-fold enhancement in two-photon excitation induced fluorescence for the typical parameter combination studied here. *J*_*e*_ = *J*_*m*_ = 10^−5^, *γ*_*m*_ = 10^−3^, *δ*_*m*_ = 1000 in equations (10) and (11), other parameters are the same as in Fig. 3.

For the uncorrelated photon pairs, the maximum amount of fluorescence is reached under optimal choices of *κ*_u.c._ and we denote this to be 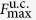. To assess whether the frequency-anticorrelated photon pairs can offer some further enhancement, we need to study the ratio of the amount of fluorescence induced by the frequency-anticorrelated photon pairs *F*^a.c.^ to the above 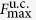. In the limit that the width of the final state |*e*〉 is very small, it is expected that only those components in the wave function with 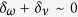 can contribute to two-photon excitation. According to this expectation, the amount of fluorescence should be roughly proportional to 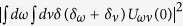. Using this expression a simple calculation then gives 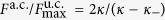, i.e., twice the ratio of the individual photon width to the sum-frequency width. In the cases that the width of the excited state (*γ*_*e*_ + 2*J*_*e*_) is not negligible, intuitively it can be viewed as a wider sum-frequency width of the incoming photon pair instead of a wider width of the excited state. This gives the estimation 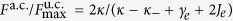 for non-negligible (*γ*_*e*_ + 2*J*_*e*_). Thus it is expected that when the width of the excited state |*e*〉 is small compared to the sum-frequency width (*κ* − *κ*_−_), the enhancement factor will reach the maximum value of 2*κ*/(*κ* − *κ*_−_), which can be quite large according to the analysis in the previous subsection. Indeed, as shown in Fig. 7 for the typical parameter combination studied here, when the width of the final state |*e*〉 is small, the frequency-anticorrelated photon pairs (equation (6)) can give a near seventy-fold enhancement over the maximum achievable amount by uncorrelated photon pairs. Also, it can be seen that the intuitive estimation 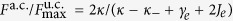 (red dashed line in Fig. 7) agrees very well with the rigorous result (black solid line in Fig. 7). Practically, this implies that for a two-photon transition with a far-detuned intermediate state, it would be more desirable to use a frequency-anticorrelated photon pair with relatively large absolute widths, so that a same width of the final state |*e*〉 will appear comparatively narrower and the enhancement factor will be larger.

## Discussion

In this paper we propose one robust, simple and general strategy “
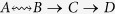
” (cf. Fig. 1) for generating frequency-anticorrrelated photon pairs. This general strategy can help guide new designs for generating frequency-anticorrelated photon pairs to reduce the required flux and thus the damage in two-photon excitation microscopy applications. We have also shown explicitly that this strategy can be realized faithfully in the coherently pumped JC model, and the photon pairs generated using the thus realized strategy can possess pronounced frequency-anticorrelation and can dramatically enhance two-photon excitation efficiency. In the derivation of the photon-pair wave function, we have employed the effective Hamiltonian *H*_eff_ instead of the original Hamiltonian *H*_pJC_. Based on the very good agreement between the predictions on the TLS-cavity dynamics given by these two Hamiltonians (cf. Fig. 3), we believe this replacement is reliable. Also, from a pragmatic point of view this replacement makes feasible the calculation, which would be formidable either analytically or numerically if the original Hamiltonian was used.

Optical two-photon lasing using strongly driven two-level systems has been studied in pioneering theoretical works^36^,^37^ and has been experimentally realized^38^. An effective Hamiltonian equivalent to equation (4) has actually been arrived in a different way in ref. 37 and its prediction has been verified experimentally^38^. Also, a conceptually similar setup^39^ has been used in generating microwave two-photon Fock state where the energy source was no longer a pumping laser but photons in a quantized cavity mode. Different from the two-photon laser, in the parameter regime studied in the present work the pair-emission is stimulated by the cavity initially in its vacuum, instead of real photons in the lasing mode. Also, as shown above, in this case it is actually possible to solve analytically for the wave function of the emitted photon pairs after including the extra-cavity modes, which enables us to investigate the photon pairs’ property and utility in detail.

In ref. 40, by following resonance features in the second-order correlation function^41^,^42^ of the cavity and studying the quantum trajectories^43^ of the TLS-cavity system, it was first pointed out that under parameter combinations similar to that studied in the present paper the coherently pumped JC model can be used to give photon-pair emission. Here the use of the effective Hamiltonian approach further clarifies the physical picture. It enables us to show that under such parameter combinations the model precisely matches our proposed general strategy for generating *frequency-anticorrelated* photon pairs. Also the use of the effective Hamiltonian and the Laplace transform method makes feasible the analytical derivation of the wave function of the outgoing photon pair carried by the *extra-cavity* photon modes, which reveals quantitatively the pronounced frequency-anticorrelation possessed by the emitted photon pairs and also enables the explicit modeling of the two-photon excitation process using the three-level model and the estimation of the enhancement in two-photon excitation induced fluorescence.

A promising candidate for realizing the discussed JC-scheme (Fig. 4) is the optical cavity QED systems with neutral atoms and Fabry-Perot type cavities^44^,^45^. In such systems the photon pair’s sum-frequency width is set by the widths of the initial and final states |−0〉 and |+0〉, and should be roughly the spontaneous emission rate of the two-level atom *γ*_TLS_. If one choose the cavity decay rate *κ* to be the same as the TLS-cavity coupling *g*, a state-of-the-art system^45^ can emit highly frequency-anticorrelated photon-pairs with a chance of 30 ~ 40%, and these photons pairs can enhance two-photon excitation efficiency by ~2*κ*/*γ*_TLS_ ~ 40 fold. For more details please see Supplementary Note 3.

For the general strategy (Fig. 1), it is probable that state A will sometimes decay directly to state D. Such a residual decay channel has two effects. Firstly, the sum-frequency width of the photon pair will be broadened to 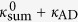, where *κ*_AD_ is the decay rate for the residual direct A → D transition while 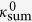 is the sum-frequency width in the absence of the residual decay. Secondly, the probability that the system goes through the desired A → B → C → D route and emit photon pairs will be reduced from unity to 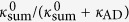. More details can be found in Supplementary Note 4.

In the future, it would be interesting to search through different physical setups for realizing the proposed general strategy and see if one of them can generate highly frequency-anticorrelated photon pairs at a high rate to enable commercialization of the use of frequency-anticorrelated photon pairs in two-photon microscopy applications. It would also be interesting to see whether there are some other simple and general strategies for the same task.

## Methods

### Explicit form of the effective Hamiltonian

The explicit expressions for the energy values appearing in the effective Hamiltonian *H*_eff_ (equation (4)) are:


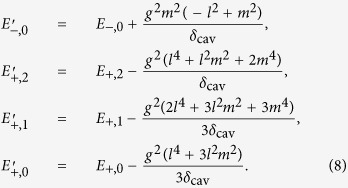


### Calculations of the evolutions of the occupation probabilities in the TLS-cavity system

In Fig. 3 we compared the occupation evolutions of the TLS-cavity system in the presence of cavity decay as predicted respectively by the original Hamiltonian 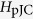 (equation (1)) and the effective Hamiltonian *H*_eff_ (equation (4)). The results of the original Hamiltonian are got by solving the master equation 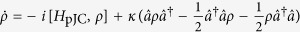
27, where *ρ* is the TLS-cavity density matrix. The occupation of any state |*s*〉 is then given by 〈*s*|*ρ*|*s*〉. The results of the effective Hamiltonian are got by solving the same master equation with *H*_pJC_ replaced by *H*_eff_.

### Sketch of the derivation of the wave function of the photon pair

Here we give a sketch of the derivation leading to the wave function of the photon pair (equation (6)) generated using the coherently pumped JC model. The general form of the wave function of the TLS-cavity plus the extra-cavity photon modes can be written as:


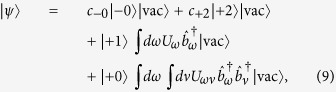


where 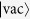 means the vacuum of the extra-cavity modes, while *c*_−0_, *c*_+2_, *U*_*ω*_ and *U*_*ωv*_ are the expansion coefficients. Note that in writing down the above wave function no terms are neglected. All terms that are not present in equation (9), such as 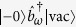, or terms containing more than 2 continuum photons, remain strictly unpopulated under *H*_effout_ (equation (5)) starting from |−0〉|vac〉. It is straightforward to write down the differential equations obeyed by the expansion coefficients according to the time-dependent Schrödinger equation. These differential equations can then be solved analytically using the Laplace transform method^19^,^46^,^47^ to give the asymptotic form (equation (6)) of the wave function of the photon pair. The relevant mathematical derivations are detailed in Supplementary Note 1. In this study we have chosen the initial state to be |−0〉|vac〉 because this is optimal and contains the essential physics. If the system is initially in |−0〉|vac〉 with probability *p* while resides in |+0〉|vac〉 with probability (1 − *p*) (as is the case if the system is initially in the ground state of the JC model 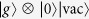), the photon pair will be generated with the probability *p* which is solely due to the initial component in |−0〉|vac〉, while the initial component in |+0〉|vac〉 will give no output.

### Sketch of the derivation of two-photon excitation induced fluorescence

In this subsection we give a sketch of the derivation of the amount of two-photon excitation induced fluorescence in the three-level model, the results of which were shown in Fig. 7. The full Hamiltonian describing the excitation and fluorescence processes is as follows (cf. Fig. 6):


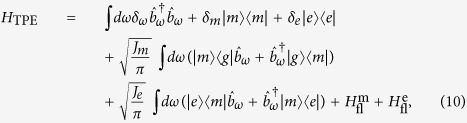


where the first term is the free energy of the continuum modes carrying the incoming photon pair (note that now the photon-pair has long left the TLS-cavity system and is impinging on the three-level system to induce two-photon excitation), the second and the third terms are the free Hamiltonians of the intermediate state |*m*〉 and the final excited state |*e*〉 respectively, the fourth and fifth terms are respectively the coupling between the 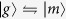 transition and the 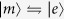 transition to the continuum modes carrying the incoming photon pair, *J*_*m*_ and *J*_*e*_ are real constants characterizing the coupling strengths, 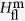 and 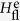 are the Hamiltonians responsible for the fluorescence from |*m*〉 and |*e*〉 respectively and are given by:


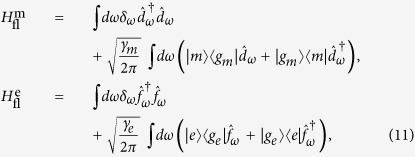


where the first terms are the free Hamiltonians of the continuum modes carrying the fluorescent photons from states |*m*〉 and |*e*〉 respectively, 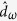 and 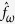 are the annihilation operators for photons in these modes and obey similar commutation relations as 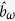, the second terms are the coupling between these continuum photon modes with the corresponding fluorescent transitions, |*g*_*m*_〉 and |*g*_*e*_〉 are the resultant states after states |*m*〉 and |*e*〉 emit the fluorescent photons, *γ*_*m*_ and *γ*_*e*_ are real constants characterizing the strength of fluorescence. In the present work we restrict ourselves to a resonant two-photon excitation. This corresponds to *δ*_*e*_ = 0 for the uncorrelated photon pair and *δ*_*e*_ = *δ*_sr_/2 (cf. equation (6)) for the frequency-anticorrelated photon pair. The general form of the wave function can be written as:


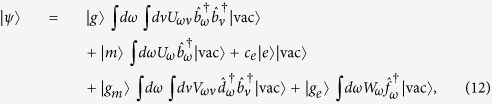


where the |vac〉 here means the vacuum of all the continuum photon modes including the modes carrying the incoming photon pair and those carrying the fluorescent photons, *U*_*ωv*_, *U*_*ω*_, *c*_*e*_, *V*_*ωv*_ and *W*_*ω*_ are expansion coefficients. It is straightforward to write down the differential equations obeyed by the expansion coefficients according to the time-dependent Schrödinger equation. Assuming the interaction strength characterized by *J*_*m*_ and *J*_*e*_ is weak, the back action on the wave function of the incoming photons can be neglected. Under this assumption the differential equations can be solved analytically using the Laplace transform method^19^,^46^,^47^ for both initial conditions corresponding respectively to the frequency-anticorrelated photon pair (equation (6)) and the uncorrelated photon pair (equation (7)). The amount of fluorescence is given by the long-time limit of the probability for occupying the fluorescent photon modes connected to the 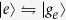 transition 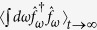, where ‘
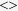
’ denotes the expectation value. The detailed mathematical derivations are given in Supplementary Note 2.

## Additional Information

**How to cite this article**: Zhang, X. *et al*. A simple and general strategy for generating frequency-anticorrelated photon pairs. *Sci. Rep*. **6**, 24509; doi: 10.1038/srep24509 (2016).

## Supplementary Material

Supplementary Information
