# Supplementary material for: A simple and general strategy for generating frequency-anticorrelated photon pairs
Source: Sci Rep. 2016 Apr 18;6:24509. doi: 10.1038/srep24509 (PMC4834487; doi:10.1038/srep24509)
Supplement: Supplementary Information [file srep24509-s1.doc]

Supplementary Information

A simple and general strategy for generating

frequency-anticorrelated photon pairs

Xin Zhang , Chang Xu, and Zhongzhou Ren

Department of Physics and Key Laboratory of Modern Acoustics,

Nanjing University, Nanjing 210008, China

Joint Center of Nuclear Science and Technology,

Nanjing University, Nanjing 210093, China

Center of Theoretical Nuclear Physics,

National Laboratory of Heavy-Ion Accelerator, Lanzhou 730000, China

Supplementary Note 1: Derivation of The Wave Function of The Photon Pair.

Here we give the detailed mathematical derivations leading to the wave function of the frequency-anticorrelated photon pair (equation (6) of the main text). The Hamiltonian is given by (equation (5) of the main text). The general form of the wave function is given by equation (9) of the main text and we reproduce it here:

The initial condition is while the initial values of all other expansion coefficients are . The time-evolution equations obeyed by the expansion coefficients under are as follows:

These equations can be solved analytically using the Laplace transform method. After Laplace transform, the above equations become:

where , , and are the Laplace transforms of , , and , respectively. From equation , can be expressed using as:

Using the above equation and equation , can be expressed in terms of as:

Using equation , can be expressed in terms of as:

Substituting equation and into equation one gets the following integral equation for :

where use has been made of . The solution of the above equation, by inspection, is:

where use has been made of . Substituting equation into equation one gets finally:

After carrying out inverse Laplace transform for the above equation and discarding transient terms, one arrives at the long-time asymptotic expression for as given in equation (6) of the main text.

Supplementary Note 2: derivation of the amount of two-photon excitation induced fluorescence.

Here we give the detailed mathematical derivations leading to the amount of two-photon excitation induced fluorescence for both the frequency-anticorrelated photon pair (equation (6) of the main text) and the uncorrelated photon pair (equation (7) of the main text), the results of which are compared in Fig 7 of the main text. The Hamiltonian is given by (equation (10) of the main text). The general form of the wave function is given by equation (12) of the main text and we reproduce it here:

the time-evolution equations obeyed by the expansion coefficients under are as follows:

where the back action on the wave function of the incoming photon pair has been dropped, according to the assumption that the interaction is sufficiently weak. Now these equations can be solved analytically using the Laplace transform method to give the amount of two-photon excitation induced fluorescence. After Laplace transform, the above equations become:

Using equations and , one can solve for in terms of , and in terms of . After substituting them back into the remaining equations , , , and using the relation one arrives at:

This equation no longer depends explicitly on the expansion coefficients for the fluorescent photon modes and . Before carrying on the solution, we shall first derive an expression for the amount of fluorescence in terms of , which we will use for calculating the final results of the amount of fluorescence. The accumulative amount of fluorescence at time is given by . The quantity we wish to calculate is . Differentiating with respect to gives:

Using equation this becomes:

According to equation , the explicit solution of in terms of is:

Substituting this into equation one gets:

where means complex conjugate. Making use of the relation the above equation becomes:

So we arrive at the following expression for the amount of two-photon excitation induced fluorescence in terms of the occupation probability of the excited level :

Now that we have this convenient formula at hand, we proceed to solve the equations - for . From equation one have:

Substituting the above equation into equation one gets:

Substituting equations and into equation one gets the following expression for in terms of the initial condition :

where use has been made of . For the uncorrelated photon pair, the initial condition is given by equation (7) of the main text. Using that equation and the above equation one gets finally:

where is the Laplace transform of the wave-function expansion coefficient for the initially uncorrelated photon pair. For the frequency-anticorrelated photon pair, the initial condition is given by equation (6) of the main text. Using that equation and equation one gets finally:

where is the Laplace transform of the wave-function expansion coefficient for the initially frequency-anticorrelated photon pair. The remaining steps are straightforward. By carrying out inverse Laplace transforms for equations and and substituting the resultant expressions for and into equation one can get the explicit analytical (somewhat lengthy) expressions for the amount of two-photon excitation induced fluorescence.

Supplementary Note 3

In this note we provide more details on the possible realization of the JC scheme using the optical cavity QED systems as mentioned in Discussion.

In Ref. 1, the realized parameters are . The experiment used two highly reflecting mirrors at the cutting edge of technology and is deep into the strong-coupling regime with . To adapt the setup to our scheme (cf. Fig. 2 of the manuscript), one of the mirrors should be made more transparent so that the cavity field will couple out dominantly through this mirror. We choose the transmission of this mirror so that the total cavity decay rate is as large as the cavity-TLS coupling .

The chance of 30~40% for the candidate system to emit photon pairs is calculated using the quantum trajectory method as in Ref. 2. This number means the system would emit photon pairs 30~40% of the time while emit single photons 60~70% of the time.

As discussed in the last part of Results, under optimal conditions the enhancement for two-photon excitation efficiency can reach twice the ratio of the individual photon width to the sum-frequency width. For the above implementation, this gives the enhancement fold.

Supplementary Note 4

In this note we investigate the effects of a residual direct A→D decay on the proposed general strategy. We model this decay as a single-photon transition from A to D. The full Hamiltonian is:

This Hamiltonian is written in a frame rotating at the reference angular frequency (cf. equation ). The first term in equation

describes the Hamiltonian evolution of the {A, B, C, D} subsystem. , , , are respectively the free energies of the states A, B, C, and D. The last term describes the interaction between states A and B. The second term in equation

describes the B→C and C→D single-photon-decay processes. , creates one photon with angular frequency , and are the decay constants for the B→C and C→D single-photon decays respectively. The third term in equation

describes the residual direct A→D single-photon decay with the decay constant . , and creates one photon with angular frequency in the photon modes related to the A→D transition. The rotating frame is defined by the reference Hamiltonian

The general form of the wave function can be written as:

where , , , and are expansion coefficients. The time-evolution equations for these coefficients under Hamiltonian equation are:

These equations can be solved analytically using the Laplace transform method. After Laplace transform, the above equations become:

From equation , one has:

Substituting this into equation one has:

Substituting this into equation one has:

From equation one has:

Substituting equations and into equation one get the following integral equation for :

The solution to this equation, by inspection, is:

Substituting equation into equation , one gets the explicit expression for . After inverse Laplace transform and discarding transient terms, one gets finally the following long-time asymptotic expression for :

From the above equation, one can see that the residual A→D decay has two effects. Firstly we shall investigate the effect of the residual decay on the sum-frequency width. The second term in the above equation can be written in the form where , , and are real numbers. The smaller one of and determines the sum-frequency width of the photon pair. For a weak coupling between states A and B, this gives, to the second order in , the sum-frequency width

For a moderate residual decay rate that is small compared to , this is essentially:

i.e., the sum-frequency width is broadened by an amount equal to the additional residual A→D decay rate.

Secondly, we shall investigate how large a fraction of A nevertheless decays to emit photon pairs in the presence of the competing A→D decay. This can be done by directly integrating in equation . Here we shall use a different method. Using equations , , and one can get the explicit expression for . After inverse Laplace transform and discarding transient terms, one gets the following long-time asymptotic expression for :

By carrying out the integration , one gets the probability for state A to decay via the A→D route:

Thus the probability for state A to decay through the desired A→B→C→D route and emit photon pairs is:

For a moderate residual decay rate that is small compared to , this is simply:

**References**

1. Hood, C. J., Lynn, T. W., Doherty, A. C., Parkins, A. S. & Kimble, H. J. The atom-cavity microscope: single atoms bound in orbit by single photons. *Science* **287,** 1447-1453 (2000).

2. Sánchez Muñoz, C. *et al.* Emitters of N-photon bundles. *Nature Photon.* **8,** 550-555 (2014).
